# Implementing digital mental health tools in the public behavioral health systems: a multi-city/county evaluation of California’s Help@Hand project

**DOI:** 10.1186/s43058-026-01053-3

**Published:** 2026-07-31

**Authors:** William Bevens, Sarah Elizabeth Stoeckl, Elizabeth Eikey, Margaret Schneider, Nicole A. Stadnick, Kai Zheng, Dana B. Mukamel, Dara H. Sorkin, Stephen M. Schueller

**Affiliations:** 1 Department of Psychology, University of California, 4341 Social and Behavioral Sciences Gateway, Irvine, CA 92697 USA; 2 Department of Psychiatry, University of California, San Diego, San Diego, CA United States; 3 Herbert Wertheim School of Public Health and Human Longevity Science, University of California, San Diego, San Diego, CA United States; 4 The Design Lab, University of California, San Diego, San Diego, CA United States; 5 Department of Public Health, University of California, Irvine, CA United States; 6 Dissemination and Implementation Science Center, UC San Diego Altman Clinical and Translational Research Institute, La Jolla, CA United States; 7 Child and Adolescent Services Research Center, San Diego, CA United States; 8 Department of Informatics, University of California, Irvine, CA United States; 9 Department of Medicine, University of California, Irvine, CA United States

**Keywords:** Digital mental health, Implementation science, Public-private partnerships, Mixed-methods evaluation, Equity, Infrastructure

## Abstract

**Background:**

Digital mental health interventions (DMHIs) can expand access to mental health care, particularly in underserved regions. Despite this, real-world implementation is complex and few studies have investigated the factors that shape it. We provide insights from Help@Hand, a five-year implementation project deploying DMHIs within public systems across counties and cities (i.e. sites) in California. This study examined how these sites implemented DMHIs within their local systems and identified factors that facilitated or prevented success.

**Methods:**

Data were collected from project leads at participating sites (*N* = 12) through four waves of surveys and two rounds of interviews. Surveys and interviews were informed by the Consolidated Framework for Implementation Research (CFIR) and were iteratively adapted across waves to capture implementation experiences at different points in the project. Interviews and surveys were analyzed using a sequential mixed-methods design. Reflexive thematic analysis and descriptive survey statistics were used to examine implementation strategies, challenges, and adaptations.

**Results:**

Integrating four surveys and two interviews, we identified seven themes whose prominence differed across project stages: (1) vendors supplemented internal expertise but offered limited customization and data access; (2) workforce challenges were common where all sites reported early labor shortages that resolved at later stages; (3) transitions to sustainment proved challenging as no sites obtained new funding at the final survey; (4) community outreach shifted toward targeted, multimodal approaches; (5) centralized project management aided contracting and coordination but introduced delays to approvals; (6) cross-site collaboration declined as the project progressed; (7) digital equity remained challenging despite greater outreach efforts reported by final project stages.

**Conclusions:**

This study provides several insights into the implementation of DMHIs within public behavioral health systems: hybrid centralized-local governance models can support coordination but may add layers of decision-making; the same determinants may be differentially experienced across sites depending on their local context; sustainment can be difficult, even with planning, where funding is difficult to obtain. We also demonstrate that the CFIR has utility in structuring implementation evaluations across multiple sites and project stages. Future multi-site DMHI initiatives should pair centralized coordination with flexible local pathways, account for differential site capacity, resource sustainment from the outset, and use frameworks such as the CFIR to structure their implementation evaluation.

**Supplementary Information:**

The online version contains supplementary material available at https://doi.org/10.1186/s43058-026-01053-3.

Contributions to the literature

- Few explorations exist of large-scale implementations of digital mental health interventions using a private-public partnership model within the United States.
- We provide mixed-methods evidence of implementation determinants experienced by counties and cities across a two-year period.
- We describe the local adaptations sites made to overcome implementation barriers, and demonstrate how the CFIR can structure evaluation across multiple sites and project stages.

## Background

Digital mental health interventions (DMHIs) are increasingly recognized for their potential to expand access to care, particularly in under-resourced or geographically isolated communities. Delivered through websites, mobile apps, text messaging, and wearable technology, DMHIs have demonstrated efficacy across a range of mental health conditions [1–5] and are often promoted as scalable solutions to workforce shortages, access gaps, and behavioral health system strain [5, 6]. These advantages led to the prioritization of research into DMHIs and a commitment to their integration within public health systems across the United States to address the growing appreciation of mental health as a public health concern [7].

Despite several notable successes within the United States demonstrating their potential [8, 9], evidence for DMHI implementation within behavioral health systems points to several recurring barriers to adoption, such as limited digital infrastructure, unclear reimbursement models, inadequate workforce capacity, and organizational misalignment, all of which converge in settings where technical resources vary widely [7–10]. Clinicians may be hesitant to adopt new technologies without clear evidence of prior success in similar settings [10]. Furthermore, agencies often lack guidance for assessing fit between digital tools and their populations, particularly in low-resource settings [11], while both clinicians and agencies often express legal concerns around privacy and confidentiality [12]. Access to devices, internet connectivity, and digital literacy all can limit populations’ ability to access DMHIs, especially among marginalized groups [13]. Beyond adoption-related barriers, sustainability is a challenge even for successful DMHI projects. Many implementation efforts languish in early stages, including pilot projects or research-driven deployments that fail to transition to sustainable system change [14, 15]. While considerations of adoption and sustainment of DMHIs from an implementation science lens exist [16], practical evidence is lacking to support behavioral health systems in bridging the gap between deployment and sustainment, particularly in public-private partnership models that span multiple sites.

California’s Help@Hand project offers a valuable opportunity to examine a DMHI implementation within the United States and identify learnings that could help inform implementations in other settings. Funded at over $80 million through the Mental Health Services Act and administered through the California Mental Health Services Authority (CalMHSA), Help@Hand represented an unprecedented statewide exploration of digital mental health. The five-year project engaged 14 county and city sites, serving nearly half of California’s 39 million residents. Sites were diverse in size, geography, and resources, ranging from urban to rural regions across the state. Despite this variation, Help@Hand promoted common learning objectives: detecting mental health needs earlier, reducing stigma, increasing access to appropriate care, promoting social connectedness, and improving data-driven service planning. As such, while implementations occurred locally within sites, common learning objectives unified sites in areas of exploration and learning related to integration of digital mental health into public behavioral health systems.

As a multi-site and multi-year initiative, Help@Hand afforded the opportunity to examine barriers to and facilitations of implementation within and between Help@Hand sites as well as their presence or absence at different times throughout the project. Using a mixed-methods approach, this study analyzed four waves of survey data and two rounds of qualitative interviews conducted over two years with site project leads to identify both site-specific and shared barriers and facilitators to implementing DMHIs across disparate, independent public behavioral systems. This study contributes to the implementation science literature by describing implementation determinants at different project stages while synthesizing insights across sites and using the frequency of reporting to reflect salience of barriers and facilitators. Thus, we contribute specific learnings that can inform subsequent implementation of mental health services within public behavioral health systems, especially DMHIs. We also advance the methodology of evaluation within implementation science by demonstrating the utility of the CFIR to guide a large-scale evaluation across multiple sites and project stages.

## Methods

### Overview

The present analysis is a sequential, mixed-methods design of implementation aspects across the Help@Hand sites. Participants were project leads directly responsible for the planning, coordination, or management of Help@Hand activities within their sites. Data collection occurred between April 2022 and November 2023 and included two rounds of semi-structured interviews and four rounds of surveys. Participating sites included City of Berkeley, Kern County, Los Angeles County, Marin County, Modoc County, Mono County, Monterey County, Orange County, Riverside County, San Francisco County, San Mateo County, Santa Barbara County, City of Tehama, and the Tri-Cities (Claremont, La Verne, and Pomona). Several sites completed their participation in Help@Hand prior to these surveys. See details in Table 1.

**Table 1:** Data collection timeline by activity and respondents

Eval Type	Administered	Reporting Period	# of Respondents
Interview 1	April – May 2022	Since April 2021	10
Survey 1	July – August 2022	Since July 2021	12
Survey 2	Oct – Dec 2022	Since Jan. 2022	12
Survey 3	March – April 2023	Since Jan. 2023	11
Interview 2	June – July 2023	Since June 2022	11
Survey 4	Oct – Nov 2023	Since Apr. 2023	8

Some sites concluded their participation in Help@Hand before the end of the overall project period, leading to fewer responses over time

### Ethics, consent, and compensation

The evaluation protocol was reviewed and approved by the University of California, Irvine Institutional Review Board. Components were designated as exempt under the “Quality Improvement Project” classification. Verbal consent, including consent to record interviews, was obtained at the beginning of each session.

### Instrument design and adaptation

Instrument development was guided by the Consolidated Framework for Implementation Research (CFIR) and refined iteratively. The evaluation team used the CFIR Interview Guide Creator to develop the initial semi-structured interview guide, selecting constructs relevant to the implementation phase across CFIR domains and adapting questions to the project context [17, 18]. The interview guide was then revised based on feedback from project collaborators and site implementation leads to support further contextual tailoring. The survey was created based on initial analysis of interview findings especially related to implementation experiences. Interview questions and survey items were iterated upon based on results of previous data collections and emerging project activities over time.

### Instrument administration

Semi-structured interviews were conducted via Zoom and typically lasted between 30 and 45 minutes. One team member led the interview while another took notes. All interviews were recorded, automatically transcribed through Zoom, and reviewed for accuracy. After each interview, the team compiled summary notes capturing initial insights and salient themes for ongoing analysis. Surveys were distributed electronically via REDCap, with follow-up reminders sent to maximize response rates. Response options included binary (e.g., “yes”/“no”) responses for successes, challenges, and accomplishments and Likert-type scales for the learnings and recommendations (e.g., “meaningful”, “very meaningful”, “moderately important”). We conducted interviews and surveys at multiple time points to both capture insights at different periods of the project and to reduce recall bias that might be introduced by reflecting over the entire project period.

## Data analysis

We employed an exploratory sequential mixed-methods design. Each data collection timepoint informed the next instrument and the materials were iterated based on analysis of the previous phase. We began with interview 1, which informed survey 1, and each subsequent phase informed the next. We analyzed qualitative and quantitative data independently before mixing and interpreting the results together.

### Interview data

Qualitative data were analyzed using reflexive thematic analysis, following Braun and Clarke’s six-phase approach [19]. Two researchers (WB and SES) independently familiarized themselves with the data by reviewing interview transcripts for accuracy, which were uploaded into ATLAS.ti for collaborative coding. Coders double-coded one transcript, then met to align codes, reconcile coding differences, and define a shared approach. Each coder analyzed a subset of transcripts independently, reconvening regularly to review coding decisions and refine the code structure (Additional File 1). Themes and sub-themes were developed inductively through discussion and initial versions were visualized using a thematic map created in Miro (Additional File 2). Subthemes were then organized by whether the underlying codes appeared at timepoint 1 only (interview 1), timepoint 2 only (interview 2), or at both timepoints. Theme refinement followed Braun and Clarke’s five-question recursive review [20], which included refining theme names, elevating or demoting themes, and combining those addressing similar constructs. All authors were members of the evaluation team, comprising university researchers independent from the managing organization and individual sites, contracted to conduct a formative evaluation across participating sites. The advantages to this structure were: 1) independence from implementation activities, reducing incentive to interpret activities as successes or failures; 2) contracted through the managing organization, which meant that researchers were not associated with any site in particular and would not elevate site-specific findings over others; 3) an external perspective that supported comparisons between sites. The disadvantages were: 1) reliance on data captured through site-partners, which contained their biases and interpretations of implementation activities; 2) reliance on periodic data collections as opposed to ongoing, periodic reflections; 3) lack of comprehensive local context to contextualize findings. These were addressed through Braun and Clarke’s five-question recursive review where researchers interrogate whether themes reflected participant accounts or evaluator assumptions [20]. In our reporting, we situate themes and subthemes with reference to specific CFIR domains and constructs. This supports comparisons with the broader literature on implementation, both generally and with respect to DMHIs.

### Survey data

Quantitative data were analyzed using descriptive statistics, including the frequency and percentage of respondents endorsing specific options (e.g., “Yes”, “Meaningful,” “Very Meaningful,” or “Agree”). Likert-type items were collapsed where appropriate to improve interpretability (e.g., combining “Meaningful,” “Moderately Meaningful,” and “Very Meaningful”). Survey data were used to identify patterns both across sites and at different times. Quantitative data were not intended for inferential testing, but provided descriptive context to complement qualitative themes. Surveys were collected at different time points to descriptively identify differences across project stages.

### Mixed-methods matrix display

To integrate qualitative and quantitative findings, we developed a matrix aligning interview data with survey data. First, themes were listed and subthemes were situated within the interview year they occurred, which could be Interview 1, 2 or both. Subthemes were then coded as a facilitator (+), barrier (-) or mixed (~) to implementation success depending on their context within themes. Then, survey questions that aligned with the themes and subthemes were situated next to them within the survey wave. Alignment of themes and survey questions was determined by researcher consensus and allowed us to explore how determinants varied over time. Some survey questions did not overlap with interview data, and were excluded from the mixed-methods analysis. The final column reported differences in frequency across surveys from both data sources. These data are described in the discussion to show how implementation dynamics differed across project phases.

## Results

### CFIR domain and construct overview

Integration of survey and interview data produced seven themes describing barriers and facilitators of DMHI implementation across the Help@Hand project. Table 2 displays the mapping of each theme to CFIR domains and constructs. All themes spanned multiple domains, which demonstrates the interplay of factors across different levels of the ecologies represented within CFIR. Inner Setting and Outer Setting constructs were the most frequently coded across subthemes, which suggests that a site’s implementation of DMHIs was greatly influenced by its organizational capacity and the broader context in which it operated. Specific to the Inner Setting, Structural Characteristics related to workforce composition, available resources and work infrastructure influenced the sustainability of workforces due to labor shortages and funding constraints. Access to Knowledge and Information was similarly relevant where staff turnover depleted institutional knowledge and limited data access from vendors impacted local decision-making. Within the Outer Setting, Partnerships and Connections were commonly coded in reference to the relationships that sites had with technology vendors, both positively and negatively. Local Conditions was another construct that repeatedly indicated the differential impact that a site’s labor market or geography had across urban or rural areas.

**Table 2:** Themes and subthemes mapped onto CFIR domains and constructs

Theme	Subtheme	CFIR Domain (Construct)
Vendors supplemented sites’ work	**+** Vendors were easy and flexible to work with.	Outer Setting (Partnerships & Connections)
	**+** Contracting streamlined sites’ work by utilizing external expertise.	Inner Setting (Access to Knowledge & Information)
	- Vendors displayed a lack of flexibility on customization.	Individuals (Implementation Facilitators)
	- Sites desired more data from the apps.	Innovation (Design); Inner Setting (Access to Knowledge & Information)
	- Difference in speed of work between sites and vendors.	Outer Setting (Partnerships & Connections); Inner Setting (Structural Characteristics)
	- Communication could be difficult between sites and vendors.	Outer Setting (Partnerships & Connections)
Sustaining a workforce proved challenging	- Lack of dedicated staffing; competing priorities and time constraints.	Inner Setting (Structural Characteristics: Work Infrastructure; Available Resources)
	- Turnover was common, and sites remained understaffed.	Outer Setting (Local Conditions); Inner Setting (Structural Characteristics: Work Infrastructure)
	- Onboarding new staff was intensive.	Individuals (Implementation Team Members)
	- Losing experienced/expert staff caused a knowledge drain.	Inner Setting (Access to Knowledge & Information); Individuals (Implementation Team Members)
Sites operated in tight funding environments	**+** Sites leveraged other resources to fill gaps.	Inner Setting (Available Resources: Funding)
	- Limited funding was available.	Outer Setting (Financing); Inner Setting (Available Resources: Funding)
	- Stakeholders scrutinized use of funding.	Outer Setting (Local Attitudes; Policies & Laws)
	**~** Sites were actively seeking funding for continuation.	Inner Setting (Readiness for Implementation)
	- Uncertainty around future innovation funding.	Outer Setting (Financing; Local Conditions)
Community outreach and marketing required new and multiple approaches	**+** In-person outreach built strong relationships.	Implementation Process (Engaging: Innovation Recipients)
	**+** Outreach involved multiple collaborations.	Outer Setting (Partnerships & Connections); Implementation Process (Teaming)
	**+** Digital equity supported outreach.	Outer Setting (Local Conditions); Individuals (Innovation Recipients: Opportunity)
	**+** Outreach involved assessing and meeting community needs.	Outer Setting (Local Attitudes; Local Conditions); Implementation Process (Planning)
	- Uncertainty around traditional marketing approaches.	Implementation Process (Engaging: Innovation Recipients)
	**+** Targeted advertising was useful at reaching specific audiences.	Implementation Process (Engaging: Innovation Recipients); Individuals (Innovation Recipients)
Project management had a central role in the project	**+** Project management supported the coordination of internal sites’ work.	Implementation Process (Planning; Teaming)
	- Communication difficulties created delays.	Outer Setting (Partnerships & Connections); Inner Setting (Relational Connections)
	- Additional layers of decision-making created complexity.	Outer Setting (Policies & Laws); Implementation Process (Engaging: Innovation Deliverers)
Collaborations were both supported and disrupted by organizational factors	**+** Pooling knowledge was beneficial.	Inner Setting (Access to Knowledge & Information; Relational Connections)
	**+** Pooling resources helped to overcome shared challenges.	Inner Setting (Available Resources); Implementation Process (Teaming)
	- Limited opportunities to reflect on other site processes and results.	Inner Setting (Relational Connections; Communications)
	- Leadership lack-of-buy-in, unresponsiveness or indecisiveness caused delays.	Inner Setting (Leadership Engagement; Readiness for Implementation)
	**+** Collaborating across departments supported outreach.	Implementation Process (Teaming; Engaging: Innovation Deliverers)
	- Missed opportunities for cross-support.	Inner Setting (Relational Connections)
	- Synchronizing sites’ processes was difficult.	Implementation Process (Teaming); Inner Setting (Structural Characteristics)
Digital equity was crucial for the community	**+** Digital literacy training helped individuals beyond Help@Hand.	Individuals (Innovation Recipients: Capability)
	- The digital divide was a barrier to reaching the community.	Outer Setting (Local Conditions); Individuals (Innovation Recipients: Opportunity)

Subthemes are coded as facilitators (+), barriers (−), or mixed (~). CFIR constructs are mapped at the subtheme level to identify where specific barriers and facilitators operate within the implementation context. Some subthemes map to multiple CFIR domains, reflecting the multilevel nature of implementation determinants

The most frequent Implementation Process constructs were Engaging Innovation Recipients, which described the efforts around marketing and outreach to community members, and Planning which described the needs assessment activities sites undertook and later-stage planning for sustainment. Mid-Level Leaders and Implementation Team Members were the most prevalent Individuals constructs, the former used in the context of responsiveness and project timelines while the latter was coded in relation to turnover and the displacement of knowledge. Innovation was the least represented of all the domains and only coded to describe the inadequate linguistic and cultural customization of the DMHIs sites used. Ultimately, the organizational and contextual factors around sites were the most reported factors affecting implementation while DMHIs themselves were rarely the primary source of either barrier or facilitation.

### Vendors supplemented sites’ work

Vendors were contracted by sites to supplement expertise and staffing capacity that were not available internally. They provided technical knowledge, implementation support, and staff to move projects forward, particularly during early phases. In this respect, vendors were viewed as an important source of Partnerships and Connections within the Outer Setting for filling gaps that could not be addressed within Inner Settings (i.e., sites) independently. Sites also reported reduced contracting with vendors over the study period, which coincided with fewer sites reporting challenges in executing contracts at latter stages compared to earlier stages of the project. Conversely, some technology vendors were unwilling or unable to customize products, a limitation of vendors as Implementation Facilitators, which led to challenges in these products addressing local needs. For example, many sites requested user-level data collected by technology vendors but as these data were rarely provided sites had limited Access to Knowledge and Information necessary for implementation. Similar Implementation Facilitator challenges emerged where vendor flexibility with linguistic adaptation varied. Some sites reported vendors as collaborative partners in addressing language needs, while others, particularly those serving large Spanish-speaking populations, described persistent gaps in translated or culturally adapted content that vendors were unwilling or unable to address.

Respondents emphasized that access to more, granular data from the DMHIs could have informed local decision-making, for example by identifying usage patterns or tailoring outreach to underserved groups. Workflow and communication mismatches also emerged early in the project where vendors moved faster than site approval processes, creating delays and frustration. By the end of the project, the challenge had shifted: communication between vendors and sites was less frequent, and some project leads noted difficulty receiving timely responses. Despite communication tensions, sites reported fewer difficulties with vendors and more frequently reported successes with vendors by the final project stages in both interviews and surveys. Ultimately, Structural Characteristics within Inner Settings had differential effects where sites with greater internal capacity and larger workforces were better positioned to manage vendor relationships and escalate unresolved issues, whereas smaller and more under-resourced sites were more dependent on vendor responsiveness and had less leverage to negotiate customization or data access.

### Sustaining a workforce proved challenging

Workforce challenges, such as labor shortages and staff bandwidth, were reported at higher frequencies during and immediately after the COVID-19 pandemic, but these Outer Setting Local Conditions remained persistent throughout the initiative. All sites reported workforce shortages on the surveys in the early waves, but this barrier was less frequent, although still salient in both the qualitative and quantitative data, at the latter stages of the project. Dedicated staffing, defined as personnel whose primary role was supporting the Help@Hand project, was considered integral to success across all stages and represented critical Inner Setting Structural Characteristics that supported Work Infrastructure and Available Resources. Surveys underscored the persistence of these challenges with dedicated staffing reported as a priority by almost all sites at the latter three stages of the project. Sites consistently reported struggles when staff were spread across multiple projects and responsibilities. High turnover within sites during and immediately after the COVID-19 pandemic led to a persistent “knowledge drain”, which depleted sites’ Access to Knowledge and Information as experienced staff left and positions remained vacant for long periods. Onboarding and training new team members, many of whom were simultaneously learning new skills on the job, placed additional strain on remaining staff. Although staffing pressures were less salient later in the project, survey data showed that three-quarters of sites reported limited workforce capacity in the final year, and many sites remained understaffed throughout the initiative. Structural Characteristics of the Inner Setting affected how sites experienced these pressures: smaller sites with limited staff were disproportionately affected, as the departure of even one experienced team member could eliminate the majority of local implementation knowledge. Sites in rural settings faced the additional Outer Setting challenge of a limited regional labor pool, making positions difficult to fill regardless of funding availability.

### Sites operated in tight funding environments

Sites felt constrained by the Financing available to them at all project stages, and they used resources from other projects (Medi-Cal Realignment, other mental health initiatives) to supplement activities for sustainment. Sites reported challenging Local Attitudes within the Outer Setting where community members and leadership across governments were critical of the program and scrutinized how money was being spent. In the closeout phase, sites sought funding to sustain their projects, which demonstrated Planning for continuity; however, uncertainty around the future of innovation funding in California limited opportunities for digital health. By the closeout phase, funding constraints had become acute with sites describing trade-offs between extending licenses for DMHIs, investing in marketing, or hiring staff and consultants.

The degree to which funding constraints shaped implementation activities varied by Inner Setting Structural Characteristics, such as site size and resources. Larger counties with established relationships had more pathways to draw on supplemental funding streams, while smaller and more rural sites had fewer options and described operating with little margin for trade-offs. Some sites were also more subject to public scrutiny of spending than others, which shaped how freely project leads could reallocate funds across activities such as marketing, staffing, and licensing.

## Community outreach and marketing required new and multiple approaches

Survey data indicated that approximately three-quarters of all sites reported that they outreached to members of the community who might be expected to benefit from the Help@Hand project, and approximately half of all sites outreached to organizations or partner groups. In-person was viewed as the preferred Implementation Process of Engaging Innovation Recipients, which included tabling at community events, live demonstrations, and one-on-one support. Without in-person engagement, flyers and brochures alone were viewed as insufficient. Successful outreach required understanding the needs of the community and their digital access, and needs assessments were useful Planning activities to better understand these Outer Setting Local Attitudes and Conditions. Teams described adjusting formats, locations, and timing of events (e.g., evenings/weekends) and adapting materials to meet language and literacy needs of their local communities. By the midpoint of the Help@Hand project, sites noted that taking a multimodal approach to community outreach supported equity and inclusion. Overall, survey data demonstrated that few sites reported challenges in marketing, particularly at the later stages of the project where initial uncertainty around traditional marketing approaches gave way to targeted advertising as an effective Implementation Process strategy for Engaging specific Innovation Recipients. Sites preferred media platforms like social media posts or Google ads over radio or physical advertising, especially as these types of new media allowed targeting of specific communities. Distributing devices or offering hands-on technology support was a useful form of outreach, especially to build relationships for further engagement.

The capacity to implement these outreach strategies varied by the Structural Characteristics within each site. Urban sites with established community partnerships and social media infrastructure were able to deploy targeted digital advertising and large-scale events, while rural sites faced Outer Setting constraints, including geographic constraints, that made in-person outreach both more logistically demanding and more essential. Sites serving highly diverse populations with significant non-English-speaking communities described additional layers of complexity in adapting materials and staffing outreach for linguistic and cultural relevance.

### Project management had a central role in the project

Respondents reported that the centralized project management entity supported Implementation Process Planning and Teaming by helping sites navigate complex contracting processes and coordinate with external vendors, particularly during early implementation. This was especially valuable for sites with limited experience in digital procurement. However, early in the project, project management’s centrality created difficulties by adding an extra layer of bureaucracy, including data sharing agreements and compliance regulations that reflected the complicated environment of Policies and Laws in the Outer Setting. Surveys reflected this ambivalence across the project timeline with respondents identifying project management as both a source of coordination and of delays. Communication challenges related to budgets and documentation were also reported throughout the project where strains in Outer Setting Partnerships created delays for sites. In contrast, project management was useful for coordinating meetings and tasks within sites, such as ensuring deadlines were met for reports.

### Collaborations were both supported and disrupted by organizational factors

Collaboration occurred across the Help@Hand project and within sites, but fewer sites reported cross-site collaboration as the project progressed. At Survey 2, half of all sites reported collaborations with other sites however, this dropped to approximately a quarter of sites by the end of the project, which coincided with qualitative data indicating that synchronizing work processes was particularly difficult in later stages. Within collaborations, pooling resources with other sites such as sharing unused app licenses or repurposing materials developed by other sites, was considered a useful means to draw on Available Resources within Inner Settings through the Implementation Process of Teaming. Pooling knowledge between sites was also viewed as critical, which included using the experiences of other project leads and managers, or replicating strategies others had implemented successfully; these activities strengthened Access to Knowledge and Information within sites, and Relational Connections across sites. Sites also reported a lack of opportunities to reflect on the outcomes from the work that other sites had undertaken and felt that there could have been more chances to collaborate if Relational Connections and Communications were improved within sites. This was reflected in reports from some sites at later stages that expressed the wish that they had been able to use resources from other sites to solve their own local problems. Similarly, at later stages sites described synchronizing processes, such as meetings or communication channels, was difficult across sites. Intra-site collaboration was also critical, with cross-departmental collaboration supporting important Implementation Processes, such as Engaging Innovation Deliverers and Teaming. However, unresponsiveness and indecisiveness from site leadership and poor cross-departmental communication created challenges for implementation, and this was understood to reflect a lack of cross-site buy-in and low Relative Priority for leadership within sites.

The value and experience of cross-site collaboration also differed by the Structural Characteristics within sites. Smaller sites described resource pooling, such as sharing unused licenses or reusing materials developed by other counties, as particularly critical, given that they lacked the internal resources to develop these independently. Larger sites, while also valuing collaboration, were less dependent on it for operational continuity.

### Digital equity was crucial for the community

Digital equity emerged as both a barrier and a vehicle for community support. Sites serving rural communities described geographic isolation and internet connectivity as barriers, while urban sites grappled with device affordability among marginalized populations and the cultural and linguistic limitations of available tools. These Local Conditions within the Outer Setting, such as limited broadband infrastructure and device affordability, provided material challenges to sites in how they addressed access. Variable Local Attitudes within the Outer Setting, including cultural resistance to technology and distrust of digital tools among individuals also presented significant challenges to sites in reaching vulnerable communities. The community members that sites were trying to serve also varied in their Capability and Opportunity as Innovation Recipients, which was dependent on whether individuals had prior experience with smartphones, reliable connectivity or sufficient data plans to sustain app use. Survey data indicated that digital literacy efforts grew over time with a third of all sites providing digital literacy training at earlier stages, while approximately half reported this by later stages. Sites reported that this training supported their community members beyond Help@Hand, such as in navigating online portals or creating resumes for job applications, which further improved Capability among Innovation Recipients in ways that extended beyond the immediate project.

At the Innovation level, Complexity and limited Adaptability meant that equity barriers went beyond the implementation contexts and were embedded in the technologies themselves. Many tools were not designed with linguistic or cultural inclusiveness in mind and few supported offline use or integrations with clinical systems. These barriers restricted sites’ ability to engage marginalized residents, particularly those in communities with limited broadband access, deaf or hard of hearing individuals and other marginalized groups. Sites reported mixed experiences addressing these barriers where some sites described vendors as collaborative partners while others encountered persistent gaps in tailoring despite their efforts to understand community needs. The absence of user-level data from vendors further limited sites’ ability to evaluate whether tools were being used by their target populations. The capacity to respond to equity challenges also varied across sites according to their Structural Characteristics with some pursuing large-scale structural responses such as hotspot lending programs, device distribution, and multilingual campaigns. Other sites, often constrained by county policy (Outer Setting) or limited staff (Inner Setting), were restricted to lower-intensity outreach efforts such as flyer distribution or brief community tabling.

## Survey data

Table 3 provides our mixed-methods matrix display integrating qualitative and quantitative findings. Insights identified in the surveys are organized both by themes discussed in the previous section and by timepoint in which the data was collected. As such, the use of repeated surveys across sites captures how sites characterized their implementation experiences at different stages of the project. It is worth noting that this survey data reflects observational descriptive patterns at different time points, especially as the respondents in some sites changed due to staff turnover in their respective project leads. Several barriers, facilitators and successes varied in their reported frequency across project stages. A greater frequency of sites reported the importance of dedicated staffing at later project stages with over 90% of sites reporting this in the final survey. Similarly, fewer sites reported obtaining additional funds at each successive survey (33% at Survey 1 and 0% at Survey 4). No sites reported submitting grants for new funding by the final stage, which suggests that despite planning sustainment is difficult to achieve. Sustaining collaboration over a long, multi-site initiative also proved challenging where cross-site teaming was reported by only a quarter of sites by Survey 4. Conversely, a greater frequency of sites reported that they engaged in digital equity outreach efforts by the final stages of the project compared to earlier stages (33% at Survey 1 and 75% at Survey 4). Sites reported that they engaged in community outreach and marketing efforts at similar frequencies at each survey timepoint however, the types of activities were different across project stages in response to local needs assessments.

**Table 3:** Joint display table combining interview and survey data across timepoints

Theme	Interview 1: 2022	Interview 2: 2023	Survey 1 (mid 2022)	Survey 2 (late 2022)	Survey 3 (early 2023)	Survey 4 (late 2023)	Changes across project stages
Vendors supplemented sites’ work.	+ Vendors were easy and flexible to work with. + Contracting streamlined sites’ work by utilizing external expertise not present. - Vendors displayed a lack of flexibility on customization. - Sites desired more data from the apps.		**+** Successfully executed a contract with a vendor: **10/12 sites** **~** Contracting for products required knowledge not present in current teams: **8/12 agreed** - Experienced contracting difficulties: **7/12 sites**	**+** Successfully executed a contract with a vendor: **7/12 sites** **~** Contracting for products required knowledge not present in current teams: **8/12 meaningful** - Experienced contracting difficulties: **7/12 sites**	**+** Successfully executed a contract with a vendor: **4/11 sites** **~** Contracting for products required knowledge not present in current teams: **6/11 meaningful** - Experienced contracting difficulties: **3/11 sites**	**+** Successfully executed a contract with a vendor: **6/8 sites** **~** Contracting for products required knowledge not present in current teams: **4/8 meaningful** - Experienced contracting difficulties: **4/8 sites**	Successfully executed contracts with vendors **varied** over time (S1 83% → S3 36% → S4 75%) while contracting to supplement knowledge gaps within sites became **less** frequently reported as meaningful over time (S1 67% → S4 50%). Contracting difficulties were **less** frequently reported over time (S1 58% → S3 27%). Initially difficulties were differences in work speeds between sites and vendors however, this shifted to communication difficulties later in the project. Lack of flexibility and access to app data was a **consistently** reported barrier over time.
	- There was a difference between sites and vendors in speed of work.	- Communication could be difficult between sites and vendors.					
Sustaining a workforce proved challenging.	- Sites experienced lack of dedicated staffing, resulting in competing priorities and time constraints. - Turnover was common, and sites remained understaffed. - Onboarding new staff was intensive. - Losing experienced/expert staff caused a knowledge drain.		**+** Dedicated staffing is necessary for project success: **7/12 agreed** - Experienced staff shortages: **12/12 sites**	**+** Dedicated staffing is necessary for project success: **12/12 meaningful** - Experienced staff shortages: **12/12 sites**	**+** Dedicated staffing is necessary for project success: **10/11 meaningful** - Experienced staff shortages: **5/11 sites**	**+** Dedicated staffing is necessary for project success: **8/8 meaningful** - Experienced staff shortages: **6/8 sites**	Dedicated staffing as a priority was reported with **greater** frequency over time (S1 58% agreed → S2–S4 > 90% meaningful) parallel to staff shortages being **less** frequently reported over time (S1 100% → S4 75%).
Sites operated in tight funding environments.	+ Sites leveraged other resources to fill gaps. - Limited funding was available. - Stakeholders scrutinized use of funding.		- Obtained additional funding to support sustainability: **4/12 sites**	- Obtained additional funding to support sustainability: **1/12 sites**	- Obtained additional funding to support sustainability: **1/11 sites** - Submitted a grant proposal for non-MHSA funding: **0/11 sites**	- Obtained additional funding to support sustainability: **0/8 sites** - Submitted a grant proposal for non-MHSA funding: **0/8 sites**	Despite seeking funding, **fewer** sites reported access to additional funding over time (S1 33% → S4 0%), partly due to limited availability. No sites (0%) submitted grant proposals and instead leveraged other available funds.
		~ Sites were actively seeking funding for project continuation. - Sites experienced uncertainty around future innovation funding.					
Community outreach and marketing required new and multiple approaches.	+ In-person outreach built strong relationships. + Outreach involved multiple collaborations. + Digital equity supported outreach. + Outreach involved assessing and meeting community needs. - Sites experienced uncertainty around traditional marketing approaches.			**+** Outreach to community members: **9/12 sites** **+** Outreach to organizations or partner groups: **7/12 sites** - Experienced challenges related to outreach: **3/12 sites** - Experienced challenges related to marketing: **4/12 sites**	**+** Outreach to community members: **8/11 sites** **+** Outreach to organizations or partner groups: **7/11 sites** - Experienced challenges related to outreach: **4/11 sites** - Experienced challenges related to marketing: **1/11 sites**	**+** Outreach to community members: **6/8 sites** **+** Outreach to organizations or partner groups: **4/8 sites** - Experienced challenges related to outreach: **2/8 sites** - Experienced challenges related to marketing: **3/8 sites**	Outreach to community members (S1 75% → S4 75%) and organizations (S1 58% → S4 50%) were **consistently** reported over time. Challenges associated with outreach were **consistently** reported and low over time (S1 25% → S4 25%) where sites used a range of strategies to support outreach. Marketing challenges **varied** in frequency of reporting over time (S1 33% → S2 9% → S3 38%) where uncertainty around traditional marketing gave way to more effective targeted advertising during the latter stages of the project.
		+ Targeted advertising was useful at reaching specific audiences.					
Project management had a central role in the project.	+ The project manager supported the coordination of internal sites’ work. - Communication difficulties created delays.						The centralized body served as a useful project manager for coordination across sites at all stags of the project, despite consistent communication challenges. At earlier stages, this central body created complexity in decision making due to layers of bureaucracy.
	- Additional layers of decision-making created complexity.						
Collaborations were both supported and disrupted by organizational factors.	+ Pooling knowledge was beneficial. + Pooling resources helped to overcome shared challenges. - Sites experienced limited opportunities to reflect on other site processes and results. - Departmental leadership lack-of-buy-in, unresponsiveness or indecisiveness caused delays.			**+** Had cross-site collaboration to support Help@Hand activities: **6/12 sites**	**+** Had cross-site collaboration to support Help@Hand activities: **3/11 sites**	**+** Had cross-site collaboration to support Help@Hand activities: **2/8 sites**	Sites reported the utility of cross-site collaboration for pooling knowledge and resources across project stages, particularly for outreach at later stages; however, collaboration was **less** frequently reported over time (S1 50% → S2 25%) where synchronizing processes across sites was difficult at later stages.
		+ Collaborating across departments supported outreach. - Sites experienced missed opportunities for cross-support. - Synchronizing sites’ processes was difficult.					
Digital equity was crucial for the community.	+ Digital literacy training helped individuals beyond the Help@Hand project. - The digital divide was a barrier to reaching the community.		**+** Provided digital literacy training to the community: **4/12 sites**	**+** Provided digital literacy training to the community: **6/12 sites**	**+** Provided digital literacy training to the community: **5/11 sites**	**+** Provided digital literacy training to the community: **6/8 sites**	Digital literacy training provided by sites was reported in **greater** frequency over time (33% → 75%).

Themes, subthemes, and survey questions were coded as facilitators (+), barriers (-), or mixed (~). “Sx” represents survey number

## Discussion

This multi-year, multi-site project provided a unique opportunity to explore barriers and facilitators associated with implementing DMHIs within a diverse, independent system of county and city behavioral health systems. Our data suggests that implementation was impacted by organizational, contextual and population characteristics of the sites; the vendor-site relationships, workforce stability, funding, governance, collaboration, digital equity, all interacted with individual sites differently and were reported at different frequencies across project stages. External vendors were a critical support, providing expertise to rapidly develop and deliver DMHIs for sites that lacked in-house capacity. The responsiveness of vendors, their willingness to customize, and the data access they provided also shaped what sites could implement. Differences in the pace of work between vendors and site staff was a challenge identified by sites that was less reported by the final stages. While this may reflect shifting work in the final stages, it may also indicate the ability for private/public partnerships to adapt over time to meet each other’s needs. Sites reported that vendors were easy to work with across all project stages, likely facilitating cross-sector understanding over time. This emphasis on vendor flexibility is critical for private/public partnerships where public-sector approvals and regulations can introduce significant delays. Regardless, sites appeared less reliant on vendors over time, possibly owing to greater worker availability after the COVID pandemic and growing recognition of the value of allocating dedicated staff to their behavioral health projects. Despite this shift, half of all sites still used vendors to supplement their workforce at the final project stages, likely reflecting variability in local workforce availability between small, rural counties and large, metropolitan counties where competition for tech workers is greater. Consequently, local conditions may dictate the ability for public sectors to develop their organizational capacity and transition from vendors to self-sustainability.

Staff turnover also drove knowledge drain through the loss of experienced staff who were difficult to replace. While staff shortages were reported less over time as outer context conditions improved, many sites remained understaffed throughout Help@Hand. This deficit had several downstream effects on a sites’ ability to operate. Firstly, the loss of experienced staff reduced organizational capacity by reducing those with the skills and local knowledge required to implement a DMHI in the public system. The staffing turnover also affected funding stability and relational connections, both built over time and vulnerable to short term disruptions [21]. This aligns with prior work in public schools [22] and community-based organizations [23], showing that staff turnover leads to losing local knowledge and the professional relationships needed to implement interventions within a community. The impact was disproportionately large in smaller teams with less access to replacement workers. This instability intersected with workforce composition, as previous Help@Hand analysis found Peers hired for lived experience were at risk of relapse and turnover without adequate structural support [24].

Secondly, replacing lost staff required new staff to be hired, onboarded and trained by the remaining staff. This left fewer staff able to commit time to Help@Hand activities, despite dedicated staffing being increasingly perceived as critical for success. The inability for staff to dedicate their time to delivering behavioral health interventions is a common sustainability challenge for implementation projects in public health and community-based systems [25–28]. Organizational capacity was not only a matter of staff skills and stability, but also of financial resources. Sites operated under funding constraints that shaped how much they could invest in staffing, technology, and outreach. Sites made trade-offs about where to allocate funding; for example, some had to choose between extending licenses for DMHIs, marketing, or hiring staff and consultants. Sites were especially cognizant of these funding constraints due to operating in a public health system where stakeholders often scrutinized Help@Hand’s premise and costs. This engendered apprehension in several sites who felt cautious about pursuing ideas that were not guaranteed to pan out, limiting agility and innovation in meeting community needs [29].

Despite recognizing limited funding, few sites secured additional funding, and none had submitted grants to public or philanthropic agencies. Grant craftsmanship is time-intensive and requires specialized skills sites may not have had, making it an unviable option for additional funding. Fortunately, sites leveraged other existing funding sources like Medicaid, Medi-Cal or other county funds, which assisted sustainability. While necessary short-term, lack of sustainability is a recognized pitfall for DMHI projects, which commonly stall in the early implementation phases, termed ‘pilotitis’ [21]. When sustainability is not built in early, DMHIs often fail to integrate into existing systems and scale appropriately. Prior implementation work in community-based organizations has shown the importance of equipping providers to identify and apply for funding [22]. Supporting sites through developing funding capacity might have alleviated some of the anxiety around sustainability.

Funding challenges were intertwined with governance. Help@Hand was governed through a hybrid model: centrally funded through a coordinating agency, but locally executed through individual county and city healthcare agencies. A persistent tension emerged between the initiative’s broader goals of system transformation and statewide innovation, and the realities of what sites could feasibly achieve given their own staff, vendor support, and institutional authority. This tension is common in multilevel projects where outer context priorities do not align with inner context capacity [23], and where the financial and governance model underpinning a project can unintentionally constrain local adaptation and create barriers to implementation [25]. While the centralized coordinating body offered contracting support and facilitated recurring meetings, supports were sometimes described as bureaucratic rather than enabling, adding steps to already complex processes without alleviating local burdens. Sites frequently operated independently, improvising solutions and developing their own materials, workflows, and protocols in the absence of centralized templates. In parallel, sites also overcame shared challenges by pooling resources, such as sharing unused product licenses. Similar to hybrid models in other contexts [26, 27], increased bureaucracy introduced by centralized coordination caused delays and frustration. Hybrid governance models require both coordination structures and strong local leadership buy-in, without which cross-organizational collaboration and knowledge sharing is difficult [28]. Ultimately, our findings suggest that for multi-agency projects over a large geographic area such as California, centralized coordination must be accompanied by flexible, localized pathways that support bottom-up innovation and adaptation in diverse settings [30], and may be more effective when it complements rather than duplicates local efforts.

Digital equity provided a critical lens through which sites enacted and redefined their activities. The early recognition that digital equity was a barrier reflected the needs assessment work sites undertook and sites reported delivering digital literacy trainings to their communities at greater frequency by the later. Beyond trainings, how sites addressed digital equity varied depending on their capacity, partnerships, and understanding of community needs. Some pursued robust structural efforts to reduce access barriers at scale, such as hotspot lending libraries, large-scale device distribution programs, and multilingual outreach campaigns. Others engaged in more constrained activities, including distributing flyers in a few community settings or brief outreach to underserved populations. Across the project, sites demonstrated a genuine commitment to equity, but their efforts often lacked the resources necessary to reach or sustain engagement with marginalized populations. These patterns align with other DMHI initiatives that have addressed equity by lowering structural barriers to access. Public libraries, for example, have been leveraged as trusted sites for adolescent engagement. For youth experiencing homelessness, distributing phones and providing mobile service has expanded both connectivity and access to digital interventions [31, 32]. Digital literacy training has likewise been shown to improve both uptake and mental health outcomes [33].

At the individual level, variation in Recipients’ Capability and Opportunity meant that even motivated users faced structural barriers to meaningful engagement. The implementation outcomes most affected were reach and adoption: the former constrained by device, connectivity, and literacy barriers, the latter by the linguistic and cultural limitations of available tools. The inability to obtain user-level data from vendors compounded these challenges, preventing sites from monitoring user engagement and limiting feedback loops needed to adapt strategies. Within the Help@Hand project, sites recognized the importance of multimodal outreach and increasingly found that targeted strategies, such as social media or Google ads, were especially effective for engaging overlooked communities. Our data extends the literature by demonstrating that multimodal and targeted approaches can reach linguistically and culturally diverse groups and geographically distinct and marginalized populations [34–36]. Still, outreach strategies alone could not overcome limitations embedded in the DMHIs themselves. Many tools were not designed with linguistic or cultural inclusiveness, accessibility, or offline functionality in mind, constraining how far equity goals could be realized in practice. This mismatch between off-the-shelf technology and community realities restricted sites’ ability to engage marginalized residents, particularly in communities with limited broadband access, deaf or hard of hearing individuals and other marginalized groups. These challenges reflect a broader pattern in the field: DMHIs are often not ready or available to meet the needs of diverse populations. Prior research has identified persistent gaps in Spanish-language mental health apps [37] and highlighted the limited adaptation of tools for marginalized and underserved groups [38]. As others have noted, DMHIs do not guarantee equity. In some cases, they may entrench existing disparities or create new forms of exclusion if not paired with intentional capacity-building [39]. The site differences in Help@Hand underscore that equity was not an inherent property of DMHIs, but something actively constructed through implementation.

These findings align with conceptualizations of health technologies as socio-technical assemblages: not discrete objects with fixed properties, but dynamic entities that take shape through interactions between human and non-human actors [40–43]. Our findings extend this insight to implementation itself, which can be thought of as a socio-technical assemblage produced through the interplay of sites, vendors, policies, infrastructure, and community contexts. Infrastructure, in particular, was not background but constitutive: staffing, contracting, integration, and access to analytics determined what the intervention could be. Our data also resonate with established implementation science frameworks, including the Exploration, Preparation, Implementation, and Sustainment (EPIS) framework [44] and the CFIR. Each emphasizes that implementation is contingent, multilevel, and evolving rather than predetermined. Most directly, CFIR was used to develop data collection materials and guide our interpretations, helping us identify where barriers and facilitators emerged and how multiple domains and constructs affected each simultaneously. Similarly, EPIS informs our interpretation by describing how determinants differed across phases of implementation, which in this study spanned preparation through sustainment. Future DMHI initiatives should therefore recognize and support interpretive and configurational work, rather than assuming that standardized tools or implementation models can be uniformly deployed across diverse settings.

## Limitations

While this study provides insights into DMHI implementation, several limitations should be noted. The findings are based on self-reported data from project leads, which may be subject to bias, including recall and social desirability bias. The reliance on a single informant may privilege managerial perspectives over others. A broader range of stakeholders, such as frontline staff, end users, and vendors, could have provided an alternate perspective on the themes presented. For example, vendors may have framed customization constraints as matters of contractual scope or technical feasibility rather than unwillingness. Similarly, frontline staff may have experienced different limitations due to funding constraints or perceived them differently without the outer context knowledge that project leads would have. The timing of data collection meant that data analysis reflected the latter stages of the project, and the pre-implementation/preparation stages of Help@Hand are not covered. While the survey data was collected at different time points from the project leads, in some instances staff turnover led to a different person responding for that site. Furthermore, we iteratively adapted the surveys to reflect insights from previous surveys and interviews. As such, our survey data should be interpreted as descriptive findings from observational data, which cannot reflect definitive longitudinal changes. Finally, California’s public behavioral health system operates under specific funding models, governance structures, and policy environments. These conditions may differ from those in other states, private healthcare systems, or international settings. Despite this, several of our findings are transferrable to other contexts: public/private partnerships are common in behavioral health systems across the world and our data discusses the challenges of and facilitators to these relationships; the impact that workforce turnover has on downstream processes would be common to many governmental contexts; the need for multimodal outreach to reach diverse populations; and the persistent tension between centralized coordination and local autonomy are structural dynamics that characterize large-scale implementation efforts regardless of jurisdiction.

## Conclusion

This evaluation of California’s Help@Hand initiative provides insights into the implementation of DMHIs within public behavioral health systems. Implementation was a process of socio-technical co-construction shaped by multiple, interrelated factors, and its success depended less on the properties of digital tools themselves than on the capacity of systems to sustain expertise, align funding and governance, create opportunities for learning, and embed equity into practice. This study makes several practical and methodological contributions to the field of DMHI implementation within behavioral health systems. First, a hybrid centralized-local governance model can support coordination and provide expertise for local sites however, this may come at the cost of additional layers of decision making. Second, the same determinants may be experienced differently due to a site’s local context, such as its labor market, geography and community characteristics, which may affect a site’s ability to weather staffing losses and funding challenges. Finally, sustainment proved difficult to achieve despite explicit planning as no site secured new external funding by project end, which suggests sustainment, including long-term funding models, must be considered during the planning stages of any DMHI implementation. Finally, we demonstrated that the CFIR can support a large-scale evaluation across multiple sites and project stages. This allowed determinants to be compared between sites, and to explore the salience of barriers and facilitators as they shifted across project stages. This illustrates the CFIR’s utility in the evaluation of complex DMHI initiatives where implementation is contingent on varied local contexts. Synthesizing these findings, future multi-site DMHI initiatives should pair centralized coordination with flexible local pathways, account for differential site capacity, and resource sustainment from the outset.

## Electronic supplementary material

Below is the link to the electronic supplementary material.

Supplementary Material 1

Supplementary Material 2

Supplementary Material 3

## Data Availability

Anonymized and deidentified quantitative material may be made available upon reasonable request. Access to raw qualitative data may not be possible due to the potential for participants to be revealed related to responses within text.
