# Supplementary material for: Implementing digital mental health tools in the public behavioral health systems: a multi-city/county evaluation of California’s Help@Hand project
Source: Implement Sci Commun. 2026 Jul 31;7:217. doi: 10.1186/s43058-026-01053-3 (PMC13721429; doi:10.1186/s43058-026-01053-3)
Supplement: Supplementary file 2 [file 43058_2026_1053_MOESM2_ESM.docx]

## Additional File 3

| **Theme** | **Illustrative Quotes** |
| --- | --- |
| **Vendors supplemented sites’ work** | *“Getting staff totally prepared for [the speed of vendors]... it still caught everybody off guard.” [S9_22]*  *“We still need customization. We can't just take this off-the-shelf app and implement [it] into [our] community... they weren’t willing to even entertain some customization.”* [S2_22]  *“...our work with the vendor has been kind of limited, we haven't gotten the data... I feel like we don't know for sure the impact...”* [S1_23] |
| **Workforce was difficult to sustain** | *“We had a brand new person who had never done contracts before... it had been over a year due to those workforce shortages.”* [S3_23]  *“I’m one of the few people that have stayed and endured through the different challenges of Covid and workloads and competing responsibilities.”* [S1_23]  *“You're trying to build programs while trying to deliver them at the same time.”* [S3_23] |
| **Sites operated in tight funding environments** | *“...we see commissioners talk badly about all this money wasted... statewide stakeholders talk about how we wasted money on Help@Hand, and that to me is challenging.”* [S2_22]  *“There are discussions about removing innovations from MHSA funds, so that will prevent progress.”* [S1_23] |
| **Community outreach and marketing required multiple approaches** | *“People had seen the flyer but didn’t really understand what it was about... it was probably because [of a] lack of staff and capacity to just go out and connect.”* [S8_23]  *“We worked with [a] marketing firm... that was the biggest thing that pushed it out… we don’t really have social media.”* [S10_22]  *“So, speaking of the current challenges... marketing has been one of the things... It was almost like an afterthought, because we were so focused and are a small team.”* [S4_23] |
| **Project management had a central role** | *“I think the [project management entity] has done well...at trying to keep counties engaged.”* [S4_23]  *“There were just delays... needing another approval, having another contract layer... it added time.”* [S4_22] |
| **Collaboration was facilitated and disrupted** | *“I appreciate hearing from other counties... The larger counties are doing creative things, so you get a sense of what could be possible.”* [S5_23]  *“We actually ended up splitting licenses... we were able to partner with [another county] and split... I felt that was very beneficial.”* [S6_22]  *“We don’t have enough time as counties... it seems more like a class presentation as opposed to... brainstorming about how to come up with different solutions.”* [S5_22] |
| **Digital equity was crucial** | *“If we are thinking about serving those within the public mental health system, one of the biggest problems with the technologies is actually the digital divide and digital literacy.”* [S7_22] |
