# Supplementary material for: Implementing digital mental health tools in the public behavioral health systems: a multi-city/county evaluation of California’s Help@Hand project
Source: Implement Sci Commun. 2026 Jul 31;7:217. doi: 10.1186/s43058-026-01053-3 (PMC13721429; doi:10.1186/s43058-026-01053-3)
Supplement: Supplementary file 3 [file 43058_2026_1053_MOESM1_ESM.docx]

## Additional File 2
